# Amitraz

**DOI:** 10.1107/S1600536813019764

**Published:** 2013-07-24

**Authors:** Sangjin Lee, Tae Ho Kim, Yong Woon Shin, Youngeun Jeon, Jineun Kim

**Affiliations:** a Department of Chemistry and Research Institute of Natural Sciences, Gyeongsang National University, Jinju 660-701, Republic of Korea; b Test and Analytical Laboratory, Korea Food and Drug Administration, 123-7 Yongdang-dong, Busan 608-829, Republic of Korea

## Abstract

In the asymmetric unit of the title compound {systematic name: *N*′-(2,4-di­methyl­phen­yl)-*N*-[*N*-(2,4-di­methyl­phen­yl)carbox­imido­yl]-*N*-methyl­methanimidamide}, C_19_H_23_N_3_, which is a formamidine pesticide, there are two independent and conformationally similar mol­ecules, with the dihedral angle between the mean planes of the 2,4-di­methylbenzene rings in each mol­ecule being 41.63 (6) and 42.09 (5)°. The crystal structure is stabilized by a C—H⋯N hydrogen bond, as well as weak inter­molecular C—H⋯π and π–π inter­actions [ring centroid separation = 3.7409 (15) Å], giving one-dimensional chains extending down the *b* direction.

## Related literature

For the toxicity and insecticidal properties of the title compound, see: Del Pino *et al.* (2013 ▶); Hollingworth (1976 ▶). For a related crystal structure, see: Peoples *et al.* (2012 ▶).
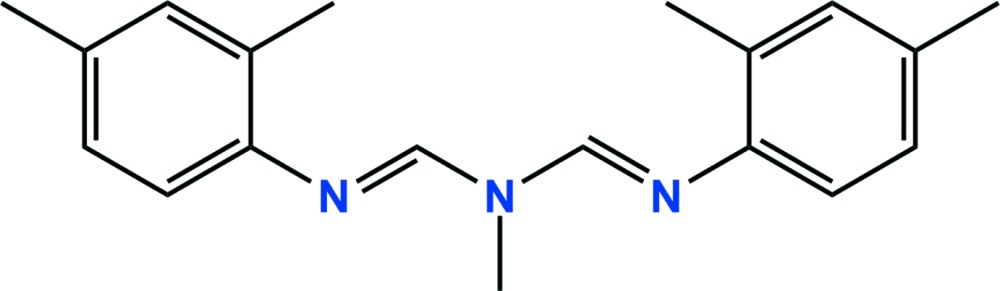


## Experimental

### 

#### Crystal data

C_19_H_23_N_3_
*M* *_r_* = 293.40
Monoclinic, 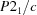

*a* = 11.9362 (11) Å
*b* = 7.5110 (7) Å
*c* = 37.514 (3) Å
β = 91.650 (2)°
*V* = 3361.9 (5) Å^3^
*Z* = 8
Mo *K*α radiation
μ = 0.07 mm^−1^
*T* = 173 K
0.40 × 0.40 × 0.40 mm

#### Data collection

Bruker APEXII CCD-detecto diffractometer
Absorption correction: multi-scan (*SADABS*; Bruker, 2006 ▶) *T*_min_ = 0.973, *T*_max_ = 0.973
18091 measured reflections
6593 independent reflections
3615 reflections with *I* > 2σ(*I*)
*R*_int_ = 0.058

#### Refinement

*R*[*F* ^2^ > 2σ(*F* ^2^)] = 0.062
*wR*(*F* ^2^) = 0.182
*S* = 0.98
6593 reflections
407 parameters
H-atom parameters constrained
Δρ_max_ = 0.26 e Å^−3^
Δρ_min_ = −0.22 e Å^−3^

### 

Data collection: *APEX2* (Bruker, 2006 ▶); cell refinement: *SAINT* (Bruker, 2006 ▶); data reduction: *SAINT*; program(s) used to solve structure: *SHELXS97* (Sheldrick, 2008 ▶); program(s) used to refine structure: *SHELXL97* (Sheldrick, 2008 ▶); molecular graphics: *SHELXTL* (Sheldrick, 2008 ▶); software used to prepare material for publication: *SHELXTL*.

## Supplementary Material

Crystal structure: contains datablock(s) global, I. DOI: 10.1107/S1600536813019764/zs2270sup1.cif

Structure factors: contains datablock(s) I. DOI: 10.1107/S1600536813019764/zs2270Isup2.hkl

Additional supplementary materials:  crystallographic information; 3D view; checkCIF report

## Figures and Tables

**Table 1:** Hydrogen-bond geometry (Å, °) *Cg*1 and *Cg*4 are the centroids of the C2–C8 and C31–C38 rings.

*D*—H⋯*A*	*D*—H	H⋯*A*	*D*⋯*A*	*D*—H⋯*A*
C29—H29*C*⋯N3^i^	0.98	2.49	3.334 (4)	144
C17—H17*A*⋯*Cg*4^i^	0.98	2.92	3.806 (3)	151
C24—H24*A*⋯*Cg*1^ii^	0.98	2.85	3.828 (3)	175
C33—H33*B*⋯*Cg*4^iii^	0.98	2.88	3.625 (3)	134

Symmetry codes: (i) 
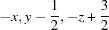
; (ii) 
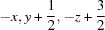
; (iii) 
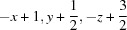
.

**Fig. 1.**
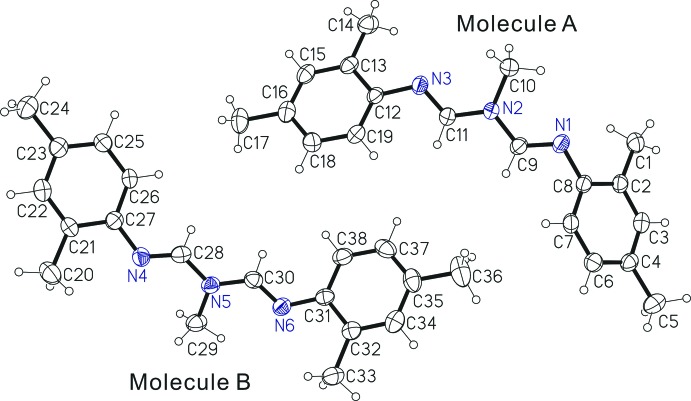
The molecular structure of the title compound, with displacement ellipsoids drawn at the 50% probability level. H atoms are shown as small spheres of arbitrary radius.

**Fig. 2.**
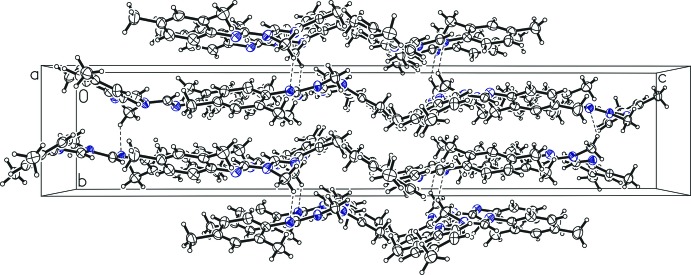
Crystal packing of the title compound in the unit cell, with weak intermolecular C—H···N hydrogen bonds shown as dashed lines.

## supplementary crystallographic information

### Comment

The title compound (C_19_H_23_N_3_) (common name amitraz) is one of the most widely used formamidine acaricide/insecticide in veterinary medicine and agriculture for use on ectoparasites (Del Pino *et al.*, 2013; Hollingworth, 1976). However, until now its crystal structure has not been reported.

In this compound (Scheme 1, Fig. 1), there are two independent and conformationally similar molecules (*A* and *B*) in the asymmetric unit, with the dihedral angle between the mean planes of the 2,4-dimethylphenyl rings in each of 41.63 (6) and 42.09 (5)°, respectively. All bond lengths and bond angles are normal and comparable to those observed in the crystal structures of a similar compound (Peoples *et al.*, 2012).

In the crystal structure (Fig. 2), an intermolecular C—H··· N hydrogen bond between a methyl C29 H atom and the N3 acceptor of the *B* molecule, as well as weak intermolecular C—H···π interactions (Table 1), are present. There is also a π–π interaction between one of the *A*-molecule 2,4-dimethylphenyl rings (defined by atoms C2–C8) [ring centroid separation 3.7409 (15) Å].

### Experimental

The title compound was purchased from the Dr Ehrenstorfer GmbH Company. Slow evaporation of a solution in CH_2_Cl_2_ gave single crystals suitable for X-ray analysis.

### Refinement

All H atoms were positioned geometrically and refined using a riding model with C—H = 0.95 Å, *U*_iso_ = 1.2*U*_eq_(C) for C*sp*^2^—H and C—H = 0.98 Å, *U*_iso_ = 1.5*U*_eq_(C) for CH_3_ groups.

### Crystal data

**Table:**

C_19_H_23_N_3_	*F*(000) = 1264
*M**_r_* = 293.40	*D*_x_ = 1.159 Mg m^−^^3^
Monoclinic, *P*2_1_/*c*	Mo *K*α radiation, λ = 0.71073 Å
Hall symbol: -P 2ybc	Cell parameters from 2173 reflections
*a* = 11.9362 (11) Å	θ = 2.7–23.1°
*b* = 7.5110 (7) Å	µ = 0.07 mm^−^^1^
*c* = 37.514 (3) Å	*T* = 173 K
β = 91.650 (2)°	Block, colourless
*V* = 3361.9 (5) Å^3^	0.40 × 0.40 × 0.40 mm
*Z* = 8	

### Data collection

**Table:**

Bruker APEXII CCD-detecto diffractometer	6593 independent reflections
Radiation source: fine-focus sealed tube	3615 reflections with *I* > 2σ(*I*)
Graphite monochromator	*R*_int_ = 0.058
φ and ω scans	θ_max_ = 26.0°, θ_min_ = 1.1°
Absorption correction: multi-scan (*SADABS*; Bruker, 2006)	*h* = −14→14
*T*_min_ = 0.973, *T*_max_ = 0.973	*k* = −9→9
18091 measured reflections	*l* = −46→36

### Refinement

**Table:**

Refinement on *F*^2^	Primary atom site location: structure-invariant direct methods
Least-squares matrix: full	Secondary atom site location: difference Fourier map
*R*[*F*^2^ > 2σ(*F*^2^)] = 0.062	Hydrogen site location: inferred from neighbouring sites
*wR*(*F*^2^) = 0.182	H-atom parameters constrained
*S* = 0.98	*w* = 1/[σ^2^(*F*_o_^2^) + (0.0907*P*)^2^] where *P* = (*F*_o_^2^ + 2*F*_c_^2^)/3
6593 reflections	(Δ/σ)_max_ = 0.001
407 parameters	Δρ_max_ = 0.26 e Å^−^^3^
0 restraints	Δρ_min_ = −0.22 e Å^−^^3^

### Special details

**Table:**

Geometry. All e.s.d.'s (except the e.s.d. in the dihedral angle between two l.s. planes) are estimated using the full covariance matrix. The cell e.s.d.'s are taken into account individually in the estimation of e.s.d.'s in distances, angles and torsion angles; correlations between e.s.d.'s in cell parameters are only used when they are defined by crystal symmetry. An approximate (isotropic) treatment of cell e.s.d.'s is used for estimating e.s.d.'s involving l.s. planes.
Refinement. Refinement of *F*^2^ against ALL reflections. The weighted *R*-factor *wR* and goodness of fit *S* are based on *F*^2^, conventional *R*-factors *R* are based on *F*, with *F* set to zero for negative *F*^2^. The threshold expression of *F*^2^ > σ(*F*^2^) is used only for calculating *R*-factors(gt) *etc*. and is not relevant to the choice of reflections for refinement. *R*-factors based on *F*^2^ are statistically about twice as large as those based on *F*, and *R*-factors based on ALL data will be even larger.

### Fractional atomic coordinates and isotropic or equivalent isotropic displacement parameters (Å^2^)

**Table:**

	*x*	*y*	*z*	*U*_iso_*/*U*_eq_	
N1	0.22503 (17)	0.8667 (3)	0.54029 (6)	0.0318 (5)	
N2	0.07334 (17)	0.8469 (3)	0.57777 (5)	0.0303 (5)	
N3	−0.06461 (17)	0.8046 (3)	0.61902 (5)	0.0316 (5)	
N4	0.02203 (18)	0.7540 (3)	0.88836 (6)	0.0349 (6)	
N5	0.16767 (17)	0.7179 (3)	0.84902 (6)	0.0335 (6)	
N6	0.30082 (18)	0.6971 (3)	0.80512 (6)	0.0342 (6)	
C1	0.3145 (2)	0.6882 (4)	0.48038 (7)	0.0393 (7)	
H1A	0.2808	0.7846	0.4661	0.059*	
H1B	0.2551	0.6175	0.4910	0.059*	
H1C	0.3591	0.6118	0.4651	0.059*	
C2	0.3887 (2)	0.7661 (3)	0.50947 (7)	0.0306 (6)	
C3	0.5052 (2)	0.7534 (3)	0.50820 (7)	0.0344 (7)	
H3	0.5367	0.6911	0.4889	0.041*	
C4	0.5770 (2)	0.8273 (3)	0.53382 (7)	0.0326 (7)	
C5	0.7027 (2)	0.8131 (4)	0.53071 (8)	0.0440 (8)	
H5A	0.7231	0.8379	0.5061	0.066*	
H5B	0.7271	0.6927	0.5373	0.066*	
H5C	0.7394	0.8997	0.5467	0.066*	
C6	0.5292 (2)	0.9178 (3)	0.56183 (7)	0.0349 (7)	
H6	0.5763	0.9703	0.5798	0.042*	
C7	0.4140 (2)	0.9327 (3)	0.56400 (7)	0.0322 (6)	
H7	0.3829	0.9970	0.5831	0.039*	
C8	0.3435 (2)	0.8542 (3)	0.53838 (7)	0.0288 (6)	
C9	0.1853 (2)	0.8345 (3)	0.57073 (7)	0.0304 (6)	
H9	0.2356	0.8002	0.5896	0.036*	
C10	−0.0063 (2)	0.9115 (4)	0.55032 (7)	0.0377 (7)	
H10A	−0.0510	0.8115	0.5410	0.057*	
H10B	0.0348	0.9660	0.5309	0.057*	
H10C	−0.0559	1.0002	0.5607	0.057*	
C11	0.0382 (2)	0.8003 (3)	0.61073 (7)	0.0317 (6)	
H11	0.0924	0.7631	0.6282	0.038*	
C12	−0.0910 (2)	0.7724 (3)	0.65503 (7)	0.0288 (6)	
C13	−0.1938 (2)	0.6914 (3)	0.66229 (7)	0.0320 (6)	
C14	−0.2710 (2)	0.6305 (4)	0.63229 (7)	0.0417 (7)	
H14A	−0.3297	0.5543	0.6418	0.063*	
H14B	−0.2281	0.5631	0.6149	0.063*	
H14C	−0.3054	0.7343	0.6206	0.063*	
C15	−0.2228 (2)	0.6670 (4)	0.69766 (7)	0.0354 (7)	
H15	−0.2925	0.6122	0.7025	0.042*	
C16	−0.1540 (2)	0.7193 (4)	0.72608 (7)	0.0372 (7)	
C17	−0.1871 (3)	0.6882 (4)	0.76443 (7)	0.0497 (8)	
H17A	−0.2529	0.6102	0.7647	0.075*	
H17B	−0.2050	0.8024	0.7755	0.075*	
H17C	−0.1248	0.6319	0.7777	0.075*	
C18	−0.0534 (2)	0.8020 (4)	0.71861 (7)	0.0393 (7)	
H18	−0.0050	0.8402	0.7377	0.047*	
C19	−0.0226 (2)	0.8295 (4)	0.68357 (7)	0.0366 (7)	
H19	0.0460	0.8881	0.6790	0.044*	
C20	−0.0414 (3)	0.9162 (4)	0.95372 (7)	0.0493 (8)	
H20A	−0.0774	0.9872	0.9721	0.074*	
H20B	0.0204	0.9843	0.9438	0.074*	
H20C	−0.0121	0.8058	0.9644	0.074*	
C21	−0.1258 (2)	0.8719 (3)	0.92448 (7)	0.0325 (6)	
C22	−0.2386 (2)	0.9098 (4)	0.92838 (7)	0.0367 (7)	
H22	−0.2612	0.9612	0.9502	0.044*	
C23	−0.3199 (2)	0.8764 (4)	0.90212 (7)	0.0345 (7)	
C24	−0.4417 (2)	0.9186 (4)	0.90765 (8)	0.0463 (8)	
H24A	−0.4476	1.0309	0.9208	0.069*	
H24B	−0.4757	0.8226	0.9213	0.069*	
H24C	−0.4809	0.9297	0.8844	0.069*	
C25	−0.2856 (2)	0.7988 (4)	0.87098 (7)	0.0395 (7)	
H25	−0.3391	0.7734	0.8524	0.047*	
C26	−0.1744 (2)	0.7576 (4)	0.86650 (7)	0.0386 (7)	
H26	−0.1530	0.7006	0.8451	0.046*	
C27	−0.0928 (2)	0.7972 (4)	0.89247 (7)	0.0322 (6)	
C28	0.0609 (2)	0.7700 (4)	0.85751 (7)	0.0343 (7)	
H28	0.0142	0.8205	0.8393	0.041*	
C29	0.2435 (2)	0.6444 (4)	0.87668 (7)	0.0399 (7)	
H29A	0.2636	0.7378	0.8940	0.060*	
H29B	0.3114	0.5997	0.8657	0.060*	
H29C	0.2060	0.5466	0.8889	0.060*	
C30	0.2031 (2)	0.7375 (3)	0.81483 (7)	0.0323 (6)	
H30	0.1516	0.7841	0.7974	0.039*	
C31	0.3252 (2)	0.7255 (3)	0.76890 (7)	0.0311 (6)	
C32	0.4296 (2)	0.8009 (3)	0.76116 (7)	0.0327 (7)	
C33	0.5113 (2)	0.8437 (4)	0.79118 (7)	0.0435 (8)	
H33A	0.4770	0.9280	0.8075	0.065*	
H33B	0.5790	0.8968	0.7815	0.065*	
H33C	0.5313	0.7342	0.8041	0.065*	
C34	0.4541 (2)	0.8329 (4)	0.72587 (8)	0.0398 (7)	
H34	0.5238	0.8867	0.7207	0.048*	
C35	0.3809 (3)	0.7896 (4)	0.69786 (8)	0.0422 (8)	
C36	0.4117 (3)	0.8261 (5)	0.65963 (8)	0.0645 (10)	
H36A	0.3974	0.7195	0.6452	0.097*	
H36B	0.4914	0.8572	0.6588	0.097*	
H36C	0.3664	0.9251	0.6502	0.097*	
C37	0.2804 (2)	0.7109 (4)	0.70560 (8)	0.0420 (8)	
H37	0.2298	0.6779	0.6867	0.050*	
C38	0.2520 (2)	0.6791 (4)	0.74065 (7)	0.0384 (7)	
H38	0.1821	0.6252	0.7455	0.046*	

### Atomic displacement parameters (Å^2^)

**Table:**

	*U*^11^	*U*^22^	*U*^33^	*U*^12^	*U*^13^	*U*^23^
N1	0.0276 (13)	0.0354 (13)	0.0327 (13)	−0.0026 (10)	0.0062 (10)	0.0000 (10)
N2	0.0257 (12)	0.0392 (13)	0.0261 (13)	0.0016 (10)	0.0039 (10)	0.0012 (10)
N3	0.0300 (13)	0.0350 (13)	0.0300 (13)	−0.0006 (10)	0.0066 (10)	0.0023 (10)
N4	0.0367 (14)	0.0377 (13)	0.0304 (13)	−0.0014 (11)	0.0025 (11)	0.0006 (11)
N5	0.0275 (13)	0.0385 (13)	0.0344 (14)	0.0012 (10)	−0.0009 (10)	0.0061 (11)
N6	0.0298 (13)	0.0364 (14)	0.0366 (14)	−0.0012 (10)	0.0025 (11)	0.0031 (11)
C1	0.0416 (17)	0.0466 (18)	0.0300 (16)	−0.0029 (14)	0.0044 (13)	−0.0033 (13)
C2	0.0316 (16)	0.0324 (15)	0.0280 (15)	−0.0010 (12)	0.0048 (12)	0.0042 (12)
C3	0.0379 (17)	0.0352 (16)	0.0307 (16)	0.0013 (13)	0.0089 (13)	0.0028 (13)
C4	0.0290 (15)	0.0313 (15)	0.0376 (17)	−0.0014 (12)	0.0033 (12)	0.0069 (13)
C5	0.0303 (16)	0.0447 (18)	0.057 (2)	0.0010 (14)	0.0034 (14)	0.0034 (15)
C6	0.0358 (16)	0.0348 (16)	0.0342 (17)	−0.0048 (13)	0.0006 (13)	0.0033 (13)
C7	0.0353 (16)	0.0362 (16)	0.0253 (15)	−0.0022 (13)	0.0062 (12)	0.0002 (12)
C8	0.0272 (14)	0.0312 (15)	0.0283 (16)	−0.0002 (12)	0.0051 (12)	0.0035 (12)
C9	0.0251 (15)	0.0335 (16)	0.0328 (16)	0.0007 (12)	0.0033 (12)	−0.0010 (12)
C10	0.0311 (16)	0.0494 (18)	0.0326 (16)	0.0064 (13)	−0.0002 (12)	0.0049 (14)
C11	0.0290 (16)	0.0383 (16)	0.0281 (15)	0.0026 (12)	0.0037 (12)	0.0004 (12)
C12	0.0237 (14)	0.0313 (15)	0.0317 (16)	0.0040 (12)	0.0039 (11)	0.0046 (12)
C13	0.0265 (15)	0.0297 (15)	0.0399 (17)	0.0041 (12)	0.0038 (12)	0.0038 (12)
C14	0.0350 (17)	0.0422 (18)	0.0478 (19)	−0.0071 (13)	0.0009 (14)	0.0069 (14)
C15	0.0310 (16)	0.0327 (16)	0.0428 (18)	0.0001 (12)	0.0095 (13)	0.0068 (13)
C16	0.0416 (17)	0.0339 (16)	0.0366 (17)	0.0085 (14)	0.0106 (14)	0.0065 (13)
C17	0.060 (2)	0.053 (2)	0.0377 (18)	0.0053 (16)	0.0165 (15)	0.0080 (15)
C18	0.0379 (17)	0.0470 (18)	0.0330 (17)	0.0050 (14)	0.0017 (13)	−0.0020 (13)
C19	0.0265 (15)	0.0461 (18)	0.0374 (17)	0.0001 (13)	0.0061 (13)	0.0016 (14)
C20	0.059 (2)	0.050 (2)	0.0384 (19)	0.0076 (16)	−0.0076 (15)	−0.0096 (15)
C21	0.0400 (17)	0.0324 (15)	0.0250 (15)	−0.0004 (13)	−0.0010 (12)	−0.0006 (12)
C22	0.0463 (18)	0.0322 (16)	0.0318 (16)	0.0014 (13)	0.0066 (14)	−0.0011 (13)
C23	0.0370 (17)	0.0328 (16)	0.0340 (17)	0.0002 (13)	0.0037 (13)	0.0068 (13)
C24	0.0432 (19)	0.0472 (19)	0.049 (2)	0.0020 (15)	0.0092 (15)	0.0045 (15)
C25	0.0348 (17)	0.0534 (19)	0.0304 (17)	−0.0037 (14)	−0.0001 (13)	0.0006 (14)
C26	0.0363 (17)	0.0537 (19)	0.0259 (16)	−0.0008 (14)	0.0055 (13)	−0.0052 (13)
C27	0.0321 (16)	0.0328 (15)	0.0319 (16)	0.0014 (12)	0.0022 (12)	0.0045 (12)
C28	0.0293 (16)	0.0348 (16)	0.0385 (17)	0.0005 (12)	−0.0029 (13)	0.0028 (13)
C29	0.0317 (16)	0.0450 (18)	0.0426 (18)	0.0012 (13)	−0.0034 (13)	0.0101 (14)
C30	0.0323 (16)	0.0323 (16)	0.0322 (16)	−0.0047 (12)	−0.0019 (12)	0.0022 (12)
C31	0.0273 (15)	0.0294 (15)	0.0364 (17)	0.0040 (12)	−0.0009 (12)	0.0000 (12)
C32	0.0313 (16)	0.0284 (15)	0.0385 (17)	0.0042 (12)	0.0012 (13)	−0.0026 (12)
C33	0.0327 (17)	0.0488 (19)	0.049 (2)	−0.0061 (14)	−0.0013 (14)	−0.0027 (15)
C34	0.0400 (18)	0.0331 (16)	0.0470 (19)	0.0034 (13)	0.0109 (15)	0.0037 (14)
C35	0.054 (2)	0.0390 (17)	0.0339 (18)	0.0128 (15)	0.0068 (15)	−0.0006 (14)
C36	0.090 (3)	0.066 (2)	0.038 (2)	0.018 (2)	0.0131 (18)	0.0051 (17)
C37	0.0427 (18)	0.0452 (18)	0.0375 (18)	0.0141 (15)	−0.0092 (14)	−0.0054 (14)
C38	0.0281 (16)	0.0424 (17)	0.0446 (19)	0.0030 (13)	−0.0011 (13)	−0.0046 (14)

### Geometric parameters (Å, º)

**Table:**

N1—C9	1.272 (3)	C16—C17	1.521 (3)
N1—C8	1.421 (3)	C17—H17A	0.9800
N2—C11	1.363 (3)	C17—H17B	0.9800
N2—C9	1.372 (3)	C17—H17C	0.9800
N2—C10	1.463 (3)	C18—C19	1.391 (3)
N3—C11	1.275 (3)	C18—H18	0.9500
N3—C12	1.417 (3)	C19—H19	0.9500
N4—C28	1.265 (3)	C20—C21	1.506 (4)
N4—C27	1.422 (3)	C20—H20A	0.9800
N5—C30	1.370 (3)	C20—H20B	0.9800
N5—C28	1.379 (3)	C20—H20C	0.9800
N5—C29	1.465 (3)	C21—C22	1.388 (4)
N6—C30	1.269 (3)	C21—C27	1.393 (3)
N6—C31	1.414 (3)	C22—C23	1.385 (4)
C1—C2	1.504 (3)	C22—H22	0.9500
C1—H1A	0.9800	C23—C25	1.378 (4)
C1—H1B	0.9800	C23—C24	1.508 (4)
C1—H1C	0.9800	C24—H24A	0.9800
C2—C8	1.393 (3)	C24—H24B	0.9800
C2—C3	1.396 (3)	C24—H24C	0.9800
C3—C4	1.385 (4)	C25—C26	1.378 (4)
C3—H3	0.9500	C25—H25	0.9500
C4—C6	1.388 (4)	C26—C27	1.389 (3)
C4—C5	1.512 (3)	C26—H26	0.9500
C5—H5A	0.9800	C28—H28	0.9500
C5—H5B	0.9800	C29—H29A	0.9800
C5—H5C	0.9800	C29—H29B	0.9800
C6—C7	1.384 (4)	C29—H29C	0.9800
C6—H6	0.9500	C30—H30	0.9500
C7—C8	1.390 (3)	C31—C38	1.399 (4)
C7—H7	0.9500	C31—C32	1.406 (4)
C9—H9	0.9500	C32—C34	1.385 (4)
C10—H10A	0.9800	C32—C33	1.503 (4)
C10—H10B	0.9800	C33—H33A	0.9800
C10—H10C	0.9800	C33—H33B	0.9800
C11—H11	0.9500	C33—H33C	0.9800
C12—C19	1.395 (4)	C34—C35	1.386 (4)
C12—C13	1.404 (3)	C34—H34	0.9500
C13—C15	1.393 (3)	C35—C37	1.377 (4)
C13—C14	1.505 (4)	C35—C36	1.516 (4)
C14—H14A	0.9800	C36—H36A	0.9800
C14—H14B	0.9800	C36—H36B	0.9800
C14—H14C	0.9800	C36—H36C	0.9800
C15—C16	1.384 (4)	C37—C38	1.388 (4)
C15—H15	0.9500	C37—H37	0.9500
C16—C18	1.388 (4)	C38—H38	0.9500
			
C9—N1—C8	115.5 (2)	C16—C18—H18	119.6
C11—N2—C9	118.9 (2)	C19—C18—H18	119.6
C11—N2—C10	120.9 (2)	C18—C19—C12	121.0 (3)
C9—N2—C10	120.2 (2)	C18—C19—H19	119.5
C11—N3—C12	118.1 (2)	C12—C19—H19	119.5
C28—N4—C27	117.3 (2)	C21—C20—H20A	109.5
C30—N5—C28	119.9 (2)	C21—C20—H20B	109.5
C30—N5—C29	120.2 (2)	H20A—C20—H20B	109.5
C28—N5—C29	119.9 (2)	C21—C20—H20C	109.5
C30—N6—C31	117.1 (2)	H20A—C20—H20C	109.5
C2—C1—H1A	109.5	H20B—C20—H20C	109.5
C2—C1—H1B	109.5	C22—C21—C27	118.2 (3)
H1A—C1—H1B	109.5	C22—C21—C20	120.6 (2)
C2—C1—H1C	109.5	C27—C21—C20	121.1 (3)
H1A—C1—H1C	109.5	C23—C22—C21	123.3 (3)
H1B—C1—H1C	109.5	C23—C22—H22	118.3
C8—C2—C3	117.9 (3)	C21—C22—H22	118.3
C8—C2—C1	121.1 (2)	C25—C23—C22	117.4 (3)
C3—C2—C1	121.0 (2)	C25—C23—C24	121.1 (3)
C4—C3—C2	123.1 (2)	C22—C23—C24	121.5 (3)
C4—C3—H3	118.5	C23—C24—H24A	109.5
C2—C3—H3	118.5	C23—C24—H24B	109.5
C3—C4—C6	117.5 (2)	H24A—C24—H24B	109.5
C3—C4—C5	120.9 (2)	C23—C24—H24C	109.5
C6—C4—C5	121.5 (3)	H24A—C24—H24C	109.5
C4—C5—H5A	109.5	H24B—C24—H24C	109.5
C4—C5—H5B	109.5	C26—C25—C23	120.6 (3)
H5A—C5—H5B	109.5	C26—C25—H25	119.7
C4—C5—H5C	109.5	C23—C25—H25	119.7
H5A—C5—H5C	109.5	C25—C26—C27	121.6 (3)
H5B—C5—H5C	109.5	C25—C26—H26	119.2
C7—C6—C4	121.0 (3)	C27—C26—H26	119.2
C7—C6—H6	119.5	C26—C27—C21	118.8 (2)
C4—C6—H6	119.5	C26—C27—N4	122.2 (2)
C6—C7—C8	120.5 (2)	C21—C27—N4	118.9 (2)
C6—C7—H7	119.7	N4—C28—N5	123.4 (3)
C8—C7—H7	119.7	N4—C28—H28	118.3
C7—C8—C2	120.0 (2)	N5—C28—H28	118.3
C7—C8—N1	121.4 (2)	N5—C29—H29A	109.5
C2—C8—N1	118.6 (2)	N5—C29—H29B	109.5
N1—C9—N2	123.4 (3)	H29A—C29—H29B	109.5
N1—C9—H9	118.3	N5—C29—H29C	109.5
N2—C9—H9	118.3	H29A—C29—H29C	109.5
N2—C10—H10A	109.5	H29B—C29—H29C	109.5
N2—C10—H10B	109.5	N6—C30—N5	123.6 (3)
H10A—C10—H10B	109.5	N6—C30—H30	118.2
N2—C10—H10C	109.5	N5—C30—H30	118.2
H10A—C10—H10C	109.5	C38—C31—C32	118.8 (3)
H10B—C10—H10C	109.5	C38—C31—N6	123.3 (2)
N3—C11—N2	122.6 (2)	C32—C31—N6	117.9 (2)
N3—C11—H11	118.7	C34—C32—C31	118.7 (3)
N2—C11—H11	118.7	C34—C32—C33	121.9 (3)
C19—C12—C13	118.7 (2)	C31—C32—C33	119.4 (2)
C19—C12—N3	122.5 (2)	C32—C33—H33A	109.5
C13—C12—N3	118.6 (2)	C32—C33—H33B	109.5
C15—C13—C12	119.0 (3)	H33A—C33—H33B	109.5
C15—C13—C14	120.6 (2)	C32—C33—H33C	109.5
C12—C13—C14	120.4 (2)	H33A—C33—H33C	109.5
C13—C14—H14A	109.5	H33B—C33—H33C	109.5
C13—C14—H14B	109.5	C32—C34—C35	122.5 (3)
H14A—C14—H14B	109.5	C32—C34—H34	118.7
C13—C14—H14C	109.5	C35—C34—H34	118.7
H14A—C14—H14C	109.5	C37—C35—C34	118.4 (3)
H14B—C14—H14C	109.5	C37—C35—C36	120.9 (3)
C16—C15—C13	122.6 (3)	C34—C35—C36	120.7 (3)
C16—C15—H15	118.7	C35—C36—H36A	109.5
C13—C15—H15	118.7	C35—C36—H36B	109.5
C15—C16—C18	118.0 (3)	H36A—C36—H36B	109.5
C15—C16—C17	121.4 (3)	C35—C36—H36C	109.5
C18—C16—C17	120.6 (3)	H36A—C36—H36C	109.5
C16—C17—H17A	109.5	H36B—C36—H36C	109.5
C16—C17—H17B	109.5	C35—C37—C38	120.8 (3)
H17A—C17—H17B	109.5	C35—C37—H37	119.6
C16—C17—H17C	109.5	C38—C37—H37	119.6
H17A—C17—H17C	109.5	C37—C38—C31	120.7 (3)
H17B—C17—H17C	109.5	C37—C38—H38	119.7
C16—C18—C19	120.7 (3)	C31—C38—H38	119.7
			
C8—C2—C3—C4	−1.4 (4)	C27—C21—C22—C23	0.2 (4)
C1—C2—C3—C4	178.2 (2)	C20—C21—C22—C23	178.3 (3)
C2—C3—C4—C6	−0.1 (4)	C21—C22—C23—C25	1.2 (4)
C2—C3—C4—C5	−178.6 (2)	C21—C22—C23—C24	179.6 (3)
C3—C4—C6—C7	0.2 (4)	C22—C23—C25—C26	−0.3 (4)
C5—C4—C6—C7	178.7 (2)	C24—C23—C25—C26	−178.7 (3)
C4—C6—C7—C8	1.1 (4)	C23—C25—C26—C27	−2.0 (4)
C6—C7—C8—C2	−2.7 (4)	C25—C26—C27—C21	3.4 (4)
C6—C7—C8—N1	179.4 (2)	C25—C26—C27—N4	179.4 (3)
C3—C2—C8—C7	2.7 (4)	C22—C21—C27—C26	−2.5 (4)
C1—C2—C8—C7	−176.8 (2)	C20—C21—C27—C26	179.5 (3)
C3—C2—C8—N1	−179.3 (2)	C22—C21—C27—N4	−178.6 (2)
C1—C2—C8—N1	1.1 (4)	C20—C21—C27—N4	3.3 (4)
C9—N1—C8—C7	−46.5 (3)	C28—N4—C27—C26	37.4 (4)
C9—N1—C8—C2	135.5 (3)	C28—N4—C27—C21	−146.6 (3)
C8—N1—C9—N2	177.4 (2)	C27—N4—C28—N5	−174.0 (2)
C11—N2—C9—N1	176.4 (2)	C30—N5—C28—N4	179.1 (2)
C10—N2—C9—N1	−4.4 (4)	C29—N5—C28—N4	−1.8 (4)
C12—N3—C11—N2	−173.2 (2)	C31—N6—C30—N5	−179.8 (2)
C9—N2—C11—N3	−178.5 (2)	C28—N5—C30—N6	178.2 (2)
C10—N2—C11—N3	2.3 (4)	C29—N5—C30—N6	−0.9 (4)
C11—N3—C12—C19	35.7 (4)	C30—N6—C31—C38	−44.3 (4)
C11—N3—C12—C13	−149.0 (2)	C30—N6—C31—C32	137.1 (3)
C19—C12—C13—C15	−1.5 (4)	C38—C31—C32—C34	2.6 (4)
N3—C12—C13—C15	−177.0 (2)	N6—C31—C32—C34	−178.8 (2)
C19—C12—C13—C14	179.0 (2)	C38—C31—C32—C33	−177.2 (2)
N3—C12—C13—C14	3.6 (4)	N6—C31—C32—C33	1.5 (4)
C12—C13—C15—C16	−0.1 (4)	C31—C32—C34—C35	−1.6 (4)
C14—C13—C15—C16	179.4 (2)	C33—C32—C34—C35	178.2 (3)
C13—C15—C16—C18	1.2 (4)	C32—C34—C35—C37	−0.4 (4)
C13—C15—C16—C17	−179.0 (2)	C32—C34—C35—C36	−179.6 (3)
C15—C16—C18—C19	−0.6 (4)	C34—C35—C37—C38	1.3 (4)
C17—C16—C18—C19	179.6 (3)	C36—C35—C37—C38	−179.4 (3)
C16—C18—C19—C12	−1.0 (4)	C35—C37—C38—C31	−0.3 (4)
C13—C12—C19—C18	2.1 (4)	C32—C31—C38—C37	−1.6 (4)
N3—C12—C19—C18	177.3 (2)	N6—C31—C38—C37	179.8 (2)

### Hydrogen-bond geometry (Å, º)

Cg1 and Cg4 are the centroids of which rings?

**Table:**

*D*—H···*A*	*D*—H	H···*A*	*D*···*A*	*D*—H···*A*
C29—H29*C*···N3^i^	0.98	2.49	3.334 (4)	144
C17—H17*A*···*Cg*4^i^	0.98	2.92	3.806 (3)	151
C24—H24*A*···*Cg*1^ii^	0.98	2.85	3.828 (3)	175
C33—H33*B*···*Cg*4^iii^	0.98	2.88	3.625 (3)	134

Symmetry codes: (i) −*x*, *y*−1/2, −*z*+3/2; (ii) −*x*, *y*+1/2, −*z*+3/2; (iii) −*x*+1, *y*+1/2, −*z*+3/2.
